# Does the Implantation of a Short-Stem Hip Prosthesis Change the Lower Limb Alignment?—Comparison of Two Modern Short-Stem Designs

**DOI:** 10.3390/jcm14072240

**Published:** 2025-03-25

**Authors:** Robert Rus, Maximilian F. Kasparek, Valerie Ladstaetter, Tobias Scheidl, Oliver Haider, Maximilian Muellner, Anna Jungwirth-Weinberger, Thomas Muellner

**Affiliations:** 1 Department of Orthopedic Surgery and Traumatology, Evangelisches Krankenhaus, 1180 Vienna, Austriatobias.scheidl@modul.at (T.S.); olihaider@gmx.at (O.H.);; 2 Clinic for Orthopaedics Paulinenhilfe, Diakonie-Klinikum Stuttgart, 70176 Stuttgart, Germany; 3 Department of Orthopaedics and Tauma Surgery, Medical University of Vienna, Vienna General Hospital, Waehringer Guertel 18-20, 1090 Vienna, Austria; 4 Center for Musculoskeletal Surgery, Charité—Universitätsmedizin Berlin, Klinik für Orthopädie, Schumannstraße 20, 10117 Berlin, Germany; maximilian.muellner@charite.de

**Keywords:** THA, short stem, limb alignment, offset, leg length

## Abstract

**Background/Objectives**: Limited data exist regarding the influence of total hip arthroplasty (THA) on the alignment of the lower limb. Therefore, the objective of this study was to investigate potential alterations in lower limb alignment (LLA) following total hip arthroplasty, with a focus on the comparison of two different short-stem implant designs. **Methods**: This retrospective study compares pre- and postoperative hip–knee–ankle angle (HKA), femoral offset, and leg length data of 115 consecutive hips with two different implant types (Mathys Optimys^®^ stem and Implantec Alpha proxy^®^ stem). **Results:** There was not a significant difference between the pre- and postoperative HKA angles regarding the entire study population (*p* = 0.293), nor after comparing the two short-stem implant designs (*p* = 0.433). Hip offset almost remained unchanged in the entire study cohort (*p* = 0.662), as well as when comparing the two short-stem implant designs (*p* = 0.206). **Conclusions**: Modern short-stem THA does not significantly affect overall LLA.

## 1. Introduction

Total hip arthroplasty (THA) is one of the most commonly performed surgical procedures in the field of orthopedics, offering significant improvements in function and quality of life for patients with osteoarthritis (OA) [1,2]. While this topic is extensively covered in orthopedic conferences and journals, and treatment approaches are continuously evolving due to the extensive research on THA, certain aspects remain less thoroughly explored. One such aspect is the impact of short-stem implant designs in THA on overall lower limb alignment (LLA). As these implants become increasingly popular due to their potential benefits, such as proximal bone stock preservation [3], there is a need to better understand how they affect not only the immediate outcomes of the surgery but also the long-term alignment of the lower extremity.

Proper lower limb alignment is crucial for maintaining joint biomechanics and ensuring an even distribution of loads across the lower extremity. Even small deviations can influence joint kinematics and may accelerate the onset or progression of OA, particularly in the knee joint [4]. THA alters the biomechanics of the proximal femur, which suggests that its effects could extend to the alignment of the entire lower limb. THA focuses mainly on factors such as acetabular cup positioning, leg length equality, and femoral offset optimization, whereas the impact on the overall LLA receives comparatively less attention pre- and interoperatively [5,6].

Several studies have investigated the impact of THA on LLA, but the findings remain inconsistent. A study by Choi et al. [6] comparing the HKA before and after THA concluded that the HKA increased from 1.4° varus to 2.7° varus postoperatively.

These findings are supported by other authors who also describe changes in the HKA in the varus direction following THA [5,6]. Van Drongelen et al. [7] also found THA to alter overall LLA and even postulated that the more varus HKA after THA could cause the progression of medial knee osteoarthritis.

Ollivier et al. [8] also reported changes in the HKA angle. However, these alterations were only minor, with no significant impact on the biomechanical parameters of the ipsilateral knee joint. Due to these inhomogeneous findings, the impact of THA on overall LLA, and the potential for these alterations to contribute to the progression of knee osteoarthritis, remains to be clarified.

However, despite the critical function of HKA in LLA, there are other crucial factors that must be considered. One such factor is leg length discrepancy (LLD). LLD is considered a prevalent cause of patient dissatisfaction following THA, leading to the necessity of its minimization without compromising prosthesis stability [9,10]. However, lower LLD is not exclusively an anatomical phenomenon, as patients frequently exhibit functional LLD. Wylde et al. [11] examined the prevalence of patient-perceived LLD post THA. Among 1114 patients, 30% reported perceiving a discrepancy in leg length. However, only a third of these patients exhibited anatomical LLD.

In recent years, short-stem femoral implants have become an increasingly popular alternative to traditional stems in THA. These implants are specifically designed to preserve more of the proximal femoral bone, promote a more natural load distribution, and offer improved adaptability to individual anatomical variations. Although their benefits have been widely documented, their effect on overall lower limb alignment remains uncertain [12,13]. Unlike conventional straight stems, short stems primarily depend on metaphyseal fixation, which may introduce greater variability in intraoperative positioning and potentially affect postoperative leg alignment. Additionally, different short-stem designs differ in their anchorage methods, alignment strategies, and reconstruction of the center of rotation, all of which may further contribute to variations in LLA [14].

As a result, the aim of this study was to analyze the potential alterations in coronal limb alignment following THA, focusing on whether short-stem designs exert an influence on LLA, and to determine the accuracy of reconstruction of LLA in two short-stem designs. By comparing two modern short-stem designs, this study aims to provide further insight into whether specific stem geometries lead to measurable differences in overall LLA and whether these variations have clinical relevance.

## 2. Materials and Methods

This retrospective data analysis includes a consecutive series of 115 hips in 108 patients who underwent primary THA with the implantation of a hip prosthesis with a short-stem design in the EKH between June 2022 and June 2023. Ethical approval by the local institutional board was obtained prior to the study (EK No. 09/2023, accepted 10 November 2023).

Patients of all sexes aged between 18 and 99 years, diagnosed with hip osteoarthritis, and meeting the surgical indication for primary total hip arthroplasty were included in this study. Additionally, patients were required to have undergone pre- and postoperative lower limb X-rays. Patients were excluded from this study if they had sustained a recent hip fracture, showed signs of osteonecrosis, had undergone revision surgery, or had previously received total knee arthroplasty (TKA) on the ipsilateral knee. Following the exclusion of ten hips that did not meet the inclusion criteria, the study cohort comprised a total of 115 hips.

**Figure 1:**
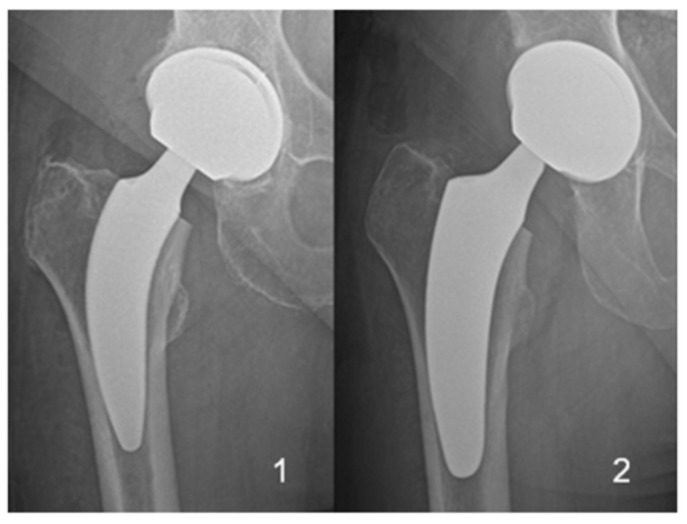
Stems compared included the Alpha Stem proxy^®^ (**1**) and Optimys^®^ (**2**).

The two different short-stem designs with proximal metaphyseal fixation, which were implanted in all patients, are shown in Figure 1. Patients were stratified into two groups based on the implanted short-stem design.

(1) ANA.NOVA Alpha stem proxy^®^ (ImplanTec GmbH, Moedling, Austria)
(2) Optimys^®^ (Mathys AG, Bettlach, Switzerland)

In a total of 44 THAs, the Alpha Stem Proxy^®^ was used, while in the other 71 THAs, the Optimys^®^ stem was utilized.

**Figure 2:**
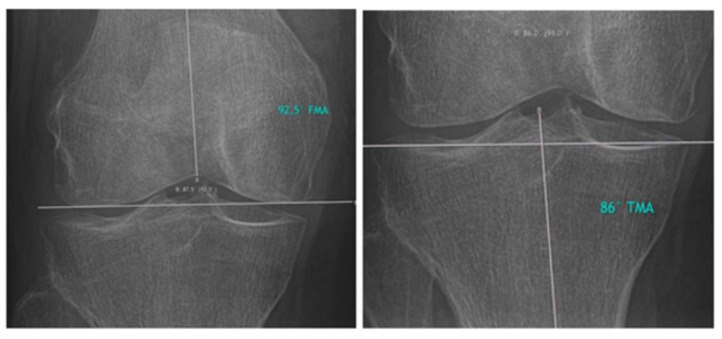
FMA and TMA measurements were performed on the THA side pre- and postoperatively.

Hirschmann et al. introduced a novel classification for phenotyping coronal lower limb alignment [15,16,17,18,19]. The measurement techniques described by Hirschmann et al. for assessing the femoral mechanical angle (FMA) and the tibial mechanical angle (TMA) were applied in this study. The measurements are presented in Figure 2.

FMA, TMA, and HKA were measured in pre- and postoperative lower limb X-rays and assessed for statistically significant discrepancies. Furthermore, potential changes in LLA were assessed by comparing the two different short-stem implant designs.

Additionally, we classified the lower limbs according to the Coronal Plane Alignment of the Knee (CPAK) classification, as previously described by MacDessi et al. [20], and the functional phenotype classification proposed by Hirschmann et al. [15].

The CPAK classification categorizes the lower limb into one of nine types based on the arithmetic HKA (aHKA) and the joint line obliquity (JLO). The aHKA is calculated by subtracting the lateral distal femoral angle (LDFA) from the FMA, while the JLO is determined by adding the LDFA and the FMA. The LDFA itself is derived by subtracting the FMA from 180°. Based on these calculations, the aHKA is classified as neutral (±2°), varus (<−2°), or valgus (>+2°), while the JLO is categorized as neutral (180° ± 3°), apex distal (<177°), or apex proximal (>183°), resulting in a total of nine CPAK types.

The functional phenotype classification assesses the FMA, TMA, and HKA in combination, grouping each phenotype within 3° intervals. A neutral (NEU) FMA was defined as 91.5° to 94.5°, a varus (VAR3°) FMA as 88.5° to 91.5°, and a valgus (VAL3°) FMA as 94.5° to 97.5°. The same intervals were applied for TMA (NEU: 85.5° to 88.5°; VAR3°: 82.5° to 85.5°; VAL3°: 88.5° to 91.5°) and HKA (NEU: 178.5° to 181.5°; VAR3°: 175.5° to 178.5°; VAL3°: 181.5° to 184.5°).

**Figure 3:**
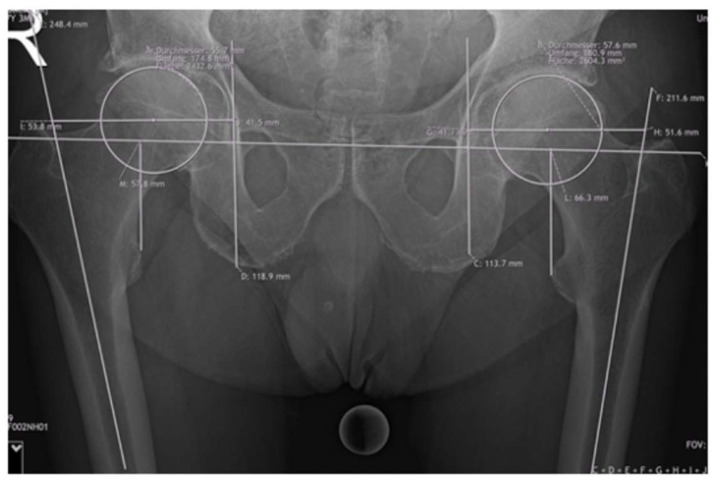
Measurements of FO, AO, HO, and leg length on a preoperative radiograph.

Offset measurements included femoral offset (FO) and acetabular offset (AO), resulting in the hip offset (HO). The FO was calculated as the perpendicular distance between the center of the femoral head and the axis of the femoral shaft. To measure acetabular offset, a vertical line was drawn through the teardrop, perpendicular to the interteardrop reference line. The perpendicular distance from this vertical line to the center of the femoral head was then measured and resulted in the AO. The LLD was assessed using the interteardrop line as the horizontal reference. The discrepancy was determined by calculating the difference between the vertical distances from this reference line to the most prominent points on each lesser trochanter. Detailed information about the measurements are shown in Figure 3.

For all patients, radiographs of the anteroposterior pelvis and lateral and lower limbs were taken pre- and postoperatively during inpatient stay. A 25 mm sphere was positioned on the inner thigh for calibration purposes. The radiographs were reviewed, and the measurements were performed by orthopedic residents and surgeons on a picture archiving and communication system (PACS) with Synedra View Professional Version 19 (Synedra Information Technologies GmbH, Innsbruck, Austria).

Two independent, blinded observers assessed the consistency of repeated measurements by analyzing 20 randomly selected radiograph sets, evaluating both intra- and interobserver reliability. The intraclass correlation coefficient (ICC) for the pre- and postoperative HKA measurements was reported as excellent (0.94 and 0.96, respectively).

Normality was assessed using the Shapiro–Wilk test. For comparisons of pre- and postoperative variables, the paired and unpaired Student’s *t*-test and the Wilcoxon signed-rank test were used accordingly. A *p*-value < 0.05 was considered statistically significant. The statistical tests were performed using IBM SPSS Statistics Version 29 (IBM Corp., Armonk, NY, USA) and Microsoft Excel.

## 3. Results

Out of 115 THAs, 72 patients were female and 43 patients were male. In 71 cases, the implant used by the surgeon was an Optimys^®^ implant, while in the remaining 44 cases, the ANA.NOVA Alpha stem proxy^®^ was used. The mean age at the time of surgery was 70 years, with a range of 43–90 years. The mean BMI was 26.7 kg/m^2^, with a range of 18.0–40.6 kg/m^2^. In 110 patients, an AMIS (Anterior Minimally Invasive Surgery) approach was used, and five patients were operated on using the ALMIS (Antero Lateral Minimally Invasive Surgery) approach. A detailed overview of the patients’ characteristics can be found in Table 1.

The mean HKA in the study cohort was comparable preoperatively (180.3°) and postoperatively (180.1°) (*p* = 0.293).

The mean FMA in the study cohort was similar preoperatively (92.6°) and postoperatively (92.4°) (*p* = 0.066). The mean TMA also remained unchanged, with preoperative (87.7°) and postoperative (87.7°) values being identical (*p* = 0.971).

The mean femoral offset in the study cohort slightly increased from 42.6 mm preoperatively to 47.1 mm postoperatively (*p* < 0.001), while the mean acetabular offset decreased from 37.3 mm preoperatively to 33.0 mm postoperatively (*p* < 0.001). However, the overall hip offset remained unchanged between 79.9 mm preoperatively and 80.1 mm postoperatively (*p* = 0.662).

When comparing the Optimys^®^ and Alpha Proxy^®^ stems, there was no significant difference in HKA (*p* = 0.433), FMA (*p* = 0.990), and TMA (*p* = 0.810). Additionally, there was no significant difference in HO (*p* = 0.206) and FO (*p* = 0.104) pre- and postoperatively when comparing the Optimys^®^ and Alpha Proxy^®^ stems. The mean AO in the Optimys^®^ group decreased from 37.7 mm preoperatively to 32.3 mm postoperatively, while the mean AO in the Alpha Proxy^®^ group decreased from 36.7 mm to 34.3 mm (*p* = 0.004).

In the entire study cohort, the mean LLD was 0.57 mm preoperatively and 1.52 mm postoperatively (*p* = 0.240). The mean LLD in the Optimys^®^ group increased from −0.49 mm preoperatively to 2.19 mm postoperatively, while the mean LLD in the Alpha Proxy^®^ group increased from 0.43 mm to 2.32 mm (*p* = 0.011).

Detailed information about the pre- and postoperative values are shown in Table 2. Detailed data of the two different short-stem implants are provided in Table 3.

The CPAK phenotype remained unchanged from the pre- to postoperative assessment in 65% (75/115) of all patients. Within the Optimys^®^ group, 61% (43/71) maintained the same CPAK phenotype, whereas in the Alpha proxy^®^ group, 74% (32/44) showed no change. A detailed overview of preoperative and postoperative CPAK phenotypes is provided in Table 4.

**Figure 4:**
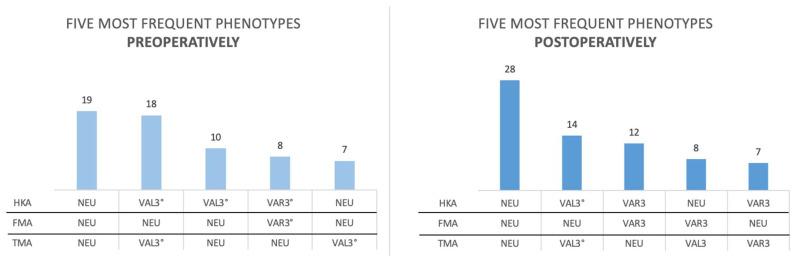
Most frequent functional knee phenotypes of all patients pre- and postoperatively; each phenotype is represented in a separate column, combining HKA, FMA, and TMA measurements. Figure 4 Legend: HKA (NEU: 178.5° to 181.5°; VAR3°: 175.5° to 178.5°; VAL3°: 181.5° to 184.5°), FMA (NEU: 91.5° to 94.5°; VAR3°: 88.5° to 91.5°; VAL3°: 94.5° to 97.5°), TMA (NEU: 85.5° to 88.5°; VAR3°: 82.5° to 85.5°; VAL3°: 88.5° to 91.5°).

According to the functional phenotype classification from Hirschmann et al. [15], the two most prevalent phenotypes remained unchanged from the pre- to postoperative assessment across all patients. Detailed results are presented in Figure 4.

## 4. Discussion

In this study, we sought to examine the impact of THA on overall limb alignment in patients utilizing a short-stem prosthesis design. The findings indicated that THA did not result in significant alterations in HKA within the study population. The lack of statistical significance in the *p*-values across the stem comparisons further reinforces the hypothesis that different prosthesis designs (e.g., Optimys^®^ vs. Alpha Proxy^®^) exert negligible influence on ipsilateral LLA. This finding suggests that stem design primarily affects local mechanics rather than global alignment.

Our findings contradict those of Choi et al., who observed an increase in mean HKA from 1.4° varus to 2.7° varus after THA, with measurements made a minimum of five years after surgery. However, given that the minimum follow-up period in this study was five years, it is plausible that the observed changes could have been influenced by knee arthritis progression. Previous studies have demonstrated that changes in coronal knee alignment following THA can favor the progression of osteoarthritis in the knee joint [21,22,23]. Aksaki et al. described that medial shifting of the hip center and the change in LLD were factors influencing the HKA and observed a mean change of 0.8° in the varus direction after THA [24]. In a separate study, Ortmaier et al. reported that patients who underwent THA with a straight stem exhibited a higher tendency to develop valgus alignment in the ipsilateral knee (57.2%) compared to those with a short stem (29%) or a native hip joint (25.8%). The degree of valgus deviation was found to be significantly higher in the straight-stem group (8.9°) compared to the short-stem (6.4°) and native joint (6.7°) groups. These effects were more pronounced in women [25]. These findings, along with our own results, suggest that short-stem THA may better preserve natural biomechanics and reduce the risk of valgus knee osteoarthritis.

Furthermore, an increase in FO from 42.6 mm preoperatively to 47.1 mm postoperatively was observed in the study cohort, alongside a decrease in AO (37.3 mm to 33.0 mm). This finding demonstrates that while individual parameters undergo shifts during THA, the total HO remains almost constant, with only a minor change from 79.9 mm to 80.1 mm. In contrast to the HO results observed in this study with short stems, van Drongelen et al. [7] examined short- and straight-stem designs and discovered that patients who received a short-stem prosthesis during THA experienced a significantly greater increase in FO (11.4 mm) compared to those with a straight-stem prosthesis (4.6 mm). Furthermore, short-stem implantation resulted in a more pronounced varus orientation of the leg (−1.4° vs. −0.4°). However, no significant difference was observed in proximal femur positioning between the two stem types. These results highlight the influence of prosthesis design on HO and leg alignment, emphasizing the need for careful preoperative planning to avoid non-physiological offset changes and deviations [26]. Boettner et al. described that in minimally invasive posterolateral THA, both standard-length and short bone-preserving stems resulted in minimal differences in HO compared to the contralateral side (0.9 mm vs. 0.1 mm) and a similar LLD (0.7 mm vs. 0.9 mm). Additionally, 86% of acetabular components were positioned within the target zone for anteversion and inclination. These findings indicate that in this surgical approach, accurate component positioning can be achieved regardless of stem type [27].

This stability in HO is crucial for postoperative hip biomechanics, as studies have shown that maintaining HO can help preserve function and balance [28,29,30]. The literature emphasizes that restoring FO during THA can prevent complications such as impingement, dislocation, and altered gait mechanics [31], which can be caused by significant deviations from the patient’s preoperative hip alignment [28,29,30,32]. Our findings suggest that despite modifications in FO and AO, the overall hip biomechanics remain unchanged, supporting a well-balanced surgical approach.

With regard to LLD, the negligible postoperative increase (from 0.57 mm to 1.52 mm) observed in this study is generally considered unproblematic, unless the discrepancy exceeds 10 mm or more. As such, small LLDs of this magnitude do not typically result in clinical symptoms or require further intervention, as postulated in other studies [9,10,11,33,34,35].

This study has several limitations. Given that it was conducted in a single center, there is a possibility that it may not be generalizable to other institutions or regions. Additionally, the involvement of patients operated on by eleven different surgeons presents a challenge in generalizing the results, as surgeon skill levels and variability in surgical techniques may have influenced the outcomes. In future studies, expanding the sample size could enhance the generalizability of the findings to larger populations and a broader range of implants.

## 5. Conclusions

In summary, the present study revealed that overall lower limb alignment (LLA) and hip offset remained stable and were not significantly affected by modern short-stem THA in general. Moreover, both examined modern short-stem THAs demonstrated similar accuracy in restoring overall lower limb alignment (LLA) and hip offset.

## Figures and Tables

**Table 1:** Patient characteristics—mean values.

	All	Optimys^®^	Alpha Proxy^®^
N	115	71	44
Sex (female/male)	(72/43)	(45/26)	(27/17)
Age (years)	70	69	72
BMI (kg/m^2^)	26.7	26.1	27.6

N = THAs in total for each group, BMI = body mass index.

**Table 2:** Mean values for HKA, FMA, TMA, HO, AO, FO, and LLD.

	Pre-OP	Post-OP	95% CI	*p*-Value
HKA (°) all	180.3	180.1	−0.50 to 0.15	0.293
HKA Optimys Group	180.9	180.7	−0.70 to 0.15	0.205
HKA Alpha proxy Group	179.2	179.2	−0.52 to 0.50	0.971
FMA (°) all	92.6	92.4	−0.37 to 0.12	0.066
FMA Optimys Group	92.9	92.6	−0.56 to −0.04	**0.023**
FMA Alpha proxy Group	92.2	92.2	−0.24 to 0.29	0.863
TMA (°) all	87.7	87.7	−0.23 to −0.24	0.971
TMA Optimys Group	88.1	88.1	−0.27 to 0.33	0.859
TMA Alpha proxy Group	87.0	87.0	−0.42 to 0.35	0.868
HO (in mm) all	79.9	80.1	−0.82 to 1.28	0.662
HO Optimys Group	81.5	81.2	−1.67 to 1.07	0.667
HO Alpha proxy Group	77.4	78.5	−0.57 to 2.73	0.194
AO (in mm) all	37.3	33.0	−5.22 to −3.35	**<0.001**
AO Optimys Group	37.7	32.3	−6.38 to −4.58	**<0.001**
AO Alpha proxy Group	36.7	34.3	−4.25 to −0.49	**0.014**
FO (in mm) all	42.6	47.1	3.49 to 5.54	**<0.001**
FO Optimys Group	43.8	48.9	3.95 to 6.42	**<0.001**
FO Alpha proxy Group	40.8	44.2	1.63 to 5.26	**<0.001**
LLD (in mm) all	0.57	1.52	−0.64 to 2.54	0.240
LLD Optimys Group	−0.49	2.19	1.01 to 4.34	**0.002**
LLD Alpha proxy Group	0.43	2.32	−4.99 to 1.21	0.226

Legend Table 2: HKA = hip–knee–ankle angle, FMA = femoral mechanical angle, TMA = tibial mechanical angle, HO = hip offset, AO = acetabular offset, FO = femoral offset, LLD = leg length discrepancy. *p* values less than 0.05 are formatted in bold.

**Table 3:** Pre- and postoperative differences in HKA, FMA, TMA, HO, AO, FO, and LLD between Optimys^®^ and Alpha proxy^®^.

Difference Between Optimys^®^ and Alpha Proxy^®^	*p*-Values	95% CI
HKA	0.433	−0.40 to 0.93
FMA	0.990	−0.71 to 0.06
TMA	0.810	−0.42 to 0.54
HO	0.206	−3.52 to 0.77
AO	**0.004**	−5.17 to −1.05
FO	0.104	−0.36 to 3.83
LLD	**0.011**	1.08 to 8.06

Legend Table 3: HKA = hip–knee–ankle angle, FMA = femoral mechanical angle, TMA = tibial mechanical angle, HO = hip offset, AO = acetabular offset, FO = femoral offset, LLD = leg length discrepancy. *p* values less than 0.05 are formatted in bold.

**Table 4:** Pre- and postoperative CPAK phenotypes based on the aHKA and the JLO of all patients in the Optimys^®^ group and the Alpha proxy^®^ group.

Optimys^®^			Alpha Proxy^®^			Optimys^®^ + Alpha Proxy^®^		
CPAK Type	Pre (n)	Post (n)	CPAK Type	Pre (n)	Post (n)	CPAK Type	Pre (n)	Post (n)
1	9	7	1	13	12	1	22	19
2	20	24	2	14	17	2	34	41
3	20	15	3	8	8	3	28	23
4	3	5	4	4	3	4	7	8
5	11	11	5	1	3	5	12	14
6	8	8	6	3	1	6	11	9
7	0	1	7	0	0	7	0	1
8	0	0	8	0	0	8	0	0
9	0	0	9	1	0	9	1	0

Table 4 Legend: Type 1: aHKA varus + JLO apex distal; Type 2: aHKA neutral + JLO apex distal; Type 3: aHKA valgus + JLO apex distal; Type 4: aHKA varus + JLO neutral; Type 5: aHKA neutral + JLO neutral; Type 6: aHKA valgus + JLO neutral; Type 7: aHKA varus + JLO apex proximal; Type 8: aHKA neutral + JLO apex proximal; Type 9: aHKA valgus + JLO apex proximal.

## Data Availability

The original contributions presented in the study are included in the article, further inquiries can be directed to the corresponding authors.
